# Impact of Sleep Deprivation on Job Performance of Working Mothers: Mediating Effect of Workplace Deviance

**DOI:** 10.3390/ijerph19073799

**Published:** 2022-03-23

**Authors:** Yuwei Deng, Jacob Cherian, Kalpina Kumari, Sarminah Samad, Jawad Abbas, Muhammad Safdar Sial, József Popp, Judit Oláh

**Affiliations:** 1 School of Mechatronics Engineering, Daqing Normal University, Daqing 163111, China; dengyuwei@dqnu.edu.cn; 2 School of Marxism, Heilongjiang University, Harbin 150080, China; 3 College of Business, Abu Dhabi University, Abu Dhabi P.O. Box 59911, United Arab Emirates; jacob.cherian@adu.ac.ae; 4 Department of Business Administration, Greenwich University, Karachi 74000, Pakistan; drkalpina@greenwich.edu.pk; 5 Department of Business Administration, College of Business and Administration, Princess Nourah Bint Abdulrahman University, Riyadh 11671, Saudi Arabia; sssamad@pnu.edu.sa; 6 Faculty of Management Sciences, University of Central Punjab, Lahore 54000, Pakistan; 7 Department of Management Sciences, COMSATS University Islamabad (CUI), Islamabad 44000, Pakistan; 8 Hungarian National Bank–Research Center, John von Neumann University, Izsáki út 10, 6000 Kecskemét, Hungary; popp.jozsef@uni-neumann.hu; 9 College of Business and Economics, University of Johannesburg, Johannesburg 2006, South Africa; olah.judit@econ.unideb.hu; 10 Faculty of Economics and Business, University of Debrecen, 4032 Debrecen, Hungary

**Keywords:** sleep deprivation, working mothers, job performance, workplace deviance

## Abstract

The current study takes its philosophical roots from organizational behavior and psychology domains to investigate the impact of sleep deprivation on the job performance of mothers working in primary, secondary, and higher education institutions (HEIs) of Pakistan. It also examines the mediating role of workplace deviance in the relationship between sleep deprivation and the job performance of working mothers. The authors followed the non-probability convenience sampling technique to study the relationship between sleep deprivation, workplace deviance, and job performance. The structural analyses indicated that sleep deprivation has a significant negative impact on the job performance of working mothers and sleep-deprived individuals often tend to perform poorly at the workplace. Such workers are also more likely to engage in workplace deviant behaviors. Moreover, workplace deviance is also found to act as a mediating variable in the relationship between sleep deprivation and job performance. The present research bridges the literature gap on the rarely investigated factors, namely sleep deprivation and workplace deviance, and provide a detailed understanding of how these factors can influence the performance of working mothers, specifically in Pakistan.

## 1. Introduction

In the modern, highly competitive business environment, organizational performance is influenced by several internal and external factors [1], such as infrastructure and facilities, financial stability, and human capital [2]. A number of researchers, for example, Pinto and He [3] and Habib et al. [4], have termed human capital as the most important element in the success or failure of any business. Organizations are established to achieve specific goals, and human resource is a driving force to achieve those goals [5,6]. With the emergence of information and communication technology (ICT), particularly the internet, the working environment of businesses has transformed a lot [7,8]. Technological development has also reshaped the needs and demands of customers [9,10].

For this reason, to meet or exceed customers’ expectations, it has become imperative for businesses to perform better than their competitors by getting the maximum output from the available resources [5,11]. In the present era, businesses face massive competition [12] and, to efficiently compete in the market, they have to review their strategies continuously [13]. The objective isn’t just to set its grounds but to remain competitive and ensure advancement in the field [14].

Similar to several other non-profit and for-profit businesses, the higher education sector has been subject to fundamental challenges in the last two decades [12]. Earlier, education was a public good by non-profit institutions with clear social purposes without commercial pressures. However, this sector has become a global service that is delivered by quasi-companies in an ever-more complex and competitive marketplace [15]. To cope with these challenges, higher education institutions need to develop an appropriate strategy to remain a competitive player in the marketplace, a necessity that is reflected in numerous calls for research on strategy in the higher education sector.

The review of the literature indicates that among all the factors, human resources has been proven as of the utmost importance and is assigned the highest value as it plays a central role in enhancing the efficiency and quality of operations [16]. An organization’s ultimate success or failure depends on its human resource. For this reason, it has become imperative for firms to investigate the factors boosting or hindering workers’ performance [17]. The literature identifies a number of factors, such as work stress, dissatisfaction with the job, lack of facilities and infrastructure, etc., that directly or indirectly impact the workers’ job performance. Sleep is another factor that can disturb the performance of individuals. According to the National Sleep Foundation [18] and Schmitt et al. [19], the role of sleep deprivation has largely been ignored by academicians and researchers concerning its impact on workers’ job performance.

The authors could not identify any study that operationalized such a hypothetical model relating to the lack of sleep, workplace deviance, and job performance of working mothers, particularly in Pakistan, where the concept of mothers as working women is rarely explored. Working mothers are the women who can combine their career with the added responsibility of raising a child and taking care of home responsibilities. They can be divided into two groups: (1) mothers working from home or (2) those who work away from home while managing to fulfill their maternal duties.

Another reason for choosing working mothers as the target population is that along with professional commitments, they have to take care of their home and family (children and spouse) irrespective of the time and situation. Both parents are frequently compelled to work because of their material aspirations and daily life needs. A well-qualified woman may demand that she be able to work and earn her own money at the same time. The single working mother is a combination of these entities, working not only to run the family but also maintaining her position as a financially independent head of the family.

Likewise, undeniably, the role of working women has changed throughout the world due to economic conditions and social demands. This has resulted in a scenario where working women have tremendous pressure to develop a professional career while sustaining active engagement in their healthy personal lives. Therefore, ever-increasing work pressure is taking a toll on working women, leaving them with less time for themselves, leading to their sleep deprivation, which can eventually destroy their performance at the workplace. For this reason, the current research aims to investigate the effect of sleep deprivation on the job performance of mothers that are working in educational institutions (schools, colleges, and universities) that are located in Pakistan. The authors took the working environment deviance as the mediating variable to examine if workplace deviance mediates the relationship between the two variables.

## 2. Theory and Literature

### 2.1. Theoretical Foundation

This theory builds it arguments on the attribution theory. The attribution theory studies the way people assign causes for their and others’ behavior [20,21]. It holistically examines all the reasons people cite for a certain success or failure that they witness in their past and how it has shaped their present-day and future attitudes. It also focuses on understanding the motivational factors behind a particular behavior and how it directly influences future successes or failures [21].

Employees are believed to be the most important asset of an organization [22]. For this reason, the management must consider the factors that might hinder the workers’ performance [23]. Dynamic firms seek individuals who give their best when performing at work. They take a greater interest in investigating the factors impacting workers’ job performance. Therefore, theoretical perspectives from psychology and organizational behavior that are supported with neurocognitive proof have been integrated into this research to examine the effects of sleep deprivation on the performance of working mothers within the educational sector of Pakistan. In particular, it has been argued that there is a negative relationship between sleep deprivation and employee performance, and the given relationship is mediated by workplace deviance. Specifically, sleep deprivation was expected to increase anxiety among employees and result in deviant behaviors at the workplace, ultimately decreasing working mothers’ overall performance. However, the conceptual framework of this research has broadly been classified into three categories, namely situational, psychological, and behavioral mechanisms. As the situational antecedent, sleep is positively correlated with workplace deviance, which makes up the psychological mechanism. This factor, in turn, negatively affects the behavioral outcome, called individual performance at the workplace.

### 2.2. Employee Performance

Every organization is established with certain objectives to be achieved [24], and those objectives are achieved by different factors of production, such as humans, land, machinery, and commodity. These factors facilitate organizations in achieving their goals [25], and, out of these factors, the human resource is the most vital [26]. It plays a central role in performing tasks for accomplishing the goals. Performance is what an organization hires people to do and do well. It should be categorically divided into actions to measure performance, i.e., an outcome and the behavior [27]. Performance is mainly measured on task performance and organizational citizenship behavior (OCB). Task performance refers to the expected behaviors and is directly involved in producing goods or services or activities that provide indirect support for the organization’s core technical processes. However, OCB refers to individual discretionary behavior, not directly or explicitly recognized by the formal reward system, and that in the aggregate promotes the effective functioning of the organization.

### 2.3. Sleep Deprivation and Employee Job Performance

According to the NSF [18], sleep is “a recurring process that keeps the individual spirits high and energizes him to embrace the daily life challenges”. It is an absolute necessity for the body’s normal functioning and to nourish the brain. Litwiller et al. [28] termed sleep as a homeostatic procedure that restores and renews the cerebrum and determines an individual’s readiness or alertness. It can also be referred to as a state of stability with extraordinarily decreased responsiveness [29] and can be separated from a trance-like state or anesthesia by its fast reversibility.

Sleep can generally be classified into two measurements: quality and quantity. The quality in sleep refers to its depth, and the quantity represents the duration. Even though sleep is essential for the smooth functioning of the human mind, many of us tend to miss out on essential hours of sleep [19]. According to the NSF [18], this phenomenon is quite common in recently becoming parents. The inadequacy of sleep leads to the inability of the brain to support the required level of wakefulness, presentation, and wellbeing in terms of health [30]. Each individual has their sleep necessity. However, for the most part, by and large, most grown-ups need around seven to eight hours of rest every night [28].

Sleep deprivation may either be partial or total. When one remains conscious for a whole night, they can add up to lack of total sleep [31]. A fractional lack of sleep is when an individual does not rest soundly and encounters frequent irregularities in sleep patterns [32,33]. Occupational demands also result in sleep deprivation, such as working in the day or night shifts, the unmanageable load of work, disorders due to sleep-loss, dependency on medication, and lifestyle changes [34]. Although partial lack of sleep is progressively predominant, some jobs even demand that individuals give up on their core hours that one can use to sleep [35], consequently adding up this factor in total or partial sleep deprivation [29].

Some researchers, such as Sivertsen et al. [31] and Bultmann et al. [36], termed disturbed sleep patterns as an important factor in employees’ absenteeism. In their study, Sivertsen et al. [31] stated that people with sleep apnoea and insomnia are highly likely to take leave for being sick. Bultmann et al. [36] conducted their study on the Danish Work Environment Cohort and said that disturbance in rest and fatigue resulted in more absent or sick leaves by employees. Rajaratnam et al. [34] also reverberated similar results in their studies.

Swanson et al. [37] examined the relationship between the work environment, sleep quality, and workers’ job performance. Specifically, they collected data from 1000 Americans working a minimum of 30 h per week by asking questions relating to the work environment, job performance, and sleep quality and found that 37% of respondents were facing sleep disorders. They concluded that extended work hours might cause chronic sleep loss leading to negative work outcomes, such as absenteeism and occupational accidents. On the other hand, employees who sleep well tend to perform better than those who lack proper sleep. Impulsive and spontaneous reactions are usually seen coming from those under sleep deprivation. They even behave socially awkwardly and resort to offensive behavior with their peers [38]. Taking into account the above discussion, to investigate how sleep deprivation impacts the performance of mothers that are working in different educational sectors of Pakistan, the following hypothesis is proposed:

**H1.**  *Sleep deprivation is a significant negative predictor of the job performance of working mothers in the educational institutions of Pakistan*.

### 2.4. Sleep Deprivation and Workplace Deviance

Business ethics are rules, standards, codes, or principles that provide guidelines for morally right behavior and truthfulness in specific situations [39]. Different people behave differently in the workplace. These behaviors inevitably affect all the workers’ activities, resulting in negative or positive outcomes. An ideal scenario at the workplace is when these behaviors are as per the code of conduct that is established by the organization [40]. Organizational norms include combinations of expected behaviors, languages, principles, and postulations that allow the workplace to perform appropriately [41]. An ideal employee realizes his responsibilities, actively performs his duties towards the organization, and fulfills his job description with justice. In addition, the employee should not be an accomplice in any mal and negligent behavior that directly or indirectly harms the interest of any individual that is associated with the organization and the organization itself [42]. However, such an ideal situation doesn’t always exist; employees may even be involved in actions that are not considered appropriate. Therefore, any destructive behavior falls under deviance [43].

Deviant workplace behavior can be defined as a collection of deliberate behaviors that harm the organization or its members. Any counterproductive work behavior, voluntary or purposeful, that functions against the organization’s passions can be termed as deviant behavior. In a workplace deviance scenario, workers intentionally violate the organizational norms that ultimately damage the performance and wellbeing of the firm [44]. This concept has taken critical importance in organizational behavior since such immoral acts that are prevalent at all levels of management [30]. For this reason, the competitive strength of different organizations remains at stake.

Workplace deviance has different forms, such as arriving late at work, not giving the best performance [45], performing personal work during the office timing, intentionally working slowly or making mistakes, etc. [46]. This sort of culpable negligence and lack of responsibility on employees is of serious concern as it can be harmful for an organization [47]. Furthermore, it may result in financial loss. It can also be an emotional loss such as mistrust and communication blockades between the customers and employees of an organization [48]. Hence, it can be concluded that acts of deviance at the workplace are consciously violating the rules and such behavior can lead to negative repercussions on the organization and its employees.

It is a common belief that the more time one invests in his work, the greater the results he would enjoy. However, in reality, this might not be the case. There is a possibility that there might be a close link between the workplace hours and deviance that is observed [49]. Irrespective of whatever position one is working in, workplace deviance creeps into all levels. Sleep-deprived senior managers often excuse the demanding nature of their job and position that they miss out on sleep [35]. The likelihood of indulging in immoral behavior, such as a low tolerance level, is highly dependent on the quantity and quality of their night’s sleep [44].

People who face sleep quality issues tend to delay things more than others [50]. Litwiller et al. [28] termed this situation as procrastination. Procrastination is due to lack of sleep, which leads to lower energy levels in a person [28]; it is inevitable when energy levels are low. The quality and quantity of rest directly affect the wellbeing, mentality, and performance at work [45]. In extraordinary conditions, the absence of rest can be a genuine and significant issue as lack of sleep can prompt the event of mishaps [51].

Sleep deprivation also results in a lack of concentration and self-control [28]. Schmitt et al. [19] and Kuhnel et al. [52] said that sleep deficiency prompts unethical and non-desired conduct in the working environment. It is because control of one’s self desires is hampered. Even a negligible amount of sleep can make a considerable difference. According to Swanson et al. [37], people who doze for six hours or less tend to get involved in undesirable work practices higher than those who doze more than six hours every night. Since this phenomenon has largely been ignored in the context of working mothers, the following hypothesis has been proposed:

**H2.**  *Sleep deprivation is a significant positive predictor of working mothers’ deviant workplace behaviors in Pakistan’s educational institutions*.

### 2.5. Workplace Deviance and Employee Job Performance

The deviant behavior in the work environment acts as a genuine danger to representative and authoritative achievement [53] and has destructive effects for obvious reasons [54]. Its impact in the workplace cannot be underestimated. It can affect many company areas, such as the ability to act decisively, efficiency and effectiveness, and the incurred monetary costs [55]. Most businesses’ downfall is the widespread deviance at the workplace, inclusive of stealing, taking undue advantage of assigned positions, and an irresponsible attitude towards quality and expenditure control [56].

Several employees can get engaged intentionally or unintentionally in immoral behaviors at the workplace, no matter their position within an organization [38]. Mawritz et al. [53] found that employees accounted for a higher percentage of retail thefts than customers. Managers are mainly concerned with deviance at the workplace [50]. The top-level managers are constantly struggling to alleviate deviant behavior from the organization for strong reasons [45]. High costs are incumbent upon organizations where workplace deviance prevails.

Managers expect their employees to perform tasks in an efficient manner [57]. The way managers perceive how their employees perform is largely influenced by workplace deviance activities, such as deceitful behavior, antagonism, and frequent absence [58]. Litzky et al. [59] termed excessive absenteeism and tardiness as withdrawal behavior. Such behavior helps workers to withdraw emotionally and physically from the firm. Chiu and Peng [60] linked the deviant behavior of workers with dishonesty, taking credit for colleagues’ work, and false accusations. They said that sometimes managers unintentionally promote deviant behavior among workers, such as personal attachments or favoritism. This may promote interpersonal deviance leading to performance deviance [46]. The deviant behaviors of employees ultimately negatively impact individual and organizational performance. Dobos [39] termed it as production deviance. Production deviance is an intentional violation of organizational standards concerning the minimum level of quantity and quality of work that is performed by workers as part of their job [39].

Dunlop and Lee [49] claimed that workplace violence costs around $4.2 billion to organizations. Moreover, employee theft costs around $200 billion, and fraudulent behavior costs around $400 billion to firms. Since this phenomenon has largely been neglected in education institutions, particularly in Pakistan, the following hypothesis has been proposed to examine it:

**H3.**  *Workplace deviance is a significant negative predictor of the job performance of working mothers in the educational institutions of Pakistan*.

One of our goals in this study is to use the perspective of attribution theory to highlight the potential consequences of sleep deprivation and specifically how it affects working mothers’ performance at the workplace. It has been argued that sleep deprivation increases workplace deviance and decreases employees’ performance. However, we also argue that workplace deviance negatively affects employees’ performance. Therefore, we believe workplace deviance represents one mechanism underlying the relationship between sleep deprivation and employees’ performance, deriving the following hypothesis;

**H4.**  *Workplace deviance mediates the relationship between sleep deprivation and the job performance of working mothers in the educational institutions of Pakistan*.

## 3. Methodology

### 3.1. Target Population and Sampling

The present research focused on working mothers from the educational sector, specifically schools, colleges, and universities in Karachi, Pakistan. Moreover, only women were eligible to participate if they were married and had given birth to one child. They are also engaged in some educational institutions (schools, colleges, and universities) for at least the last 6 months. Since it was not possible to get accurate information about the total number of working mothers in the city, the authors preferred to follow a convenience sampling technique to collect the data.

The current research took validated items from Bennett and Robinson’s [61] and Podsakoff and MacKenzie’s [62] studies (details are given in the description of measures section). A pilot study was carried out to ensure the adapted instruments’ reliability and validity. A total of 60 questionnaires were administered in 7 schools, 6 colleges, and 5 universities. Out of those 60 questionnaires, 53 filled questionnaires were returned, with a response rate of 88%. The initial survey indicated the internal consistency of the constructs with the Cronbach’s alpha value of 0.913. This provided confidence to the researchers for a detailed survey.

The rule for effective research is between 30 to 500 responses or ten times the number of variables [63]. A total of 402 questionnaires were self-administered to the employees of different educational institutions in Karachi, Pakistan. Out of 402 questionnaires, 210 were distributed to schools (response 201; response rate 96%), 120 were administered to colleges (response 108; response rate 90%), and 72 were served to universities (response 68; response rate 97%). A total of 377 filled questionnaires out of 402 were returned, representing a 94% response rate (see Table 1).

### 3.2. Data Collection Method

The current research followed an empirical approach to collect the data. At the very start, permission was sought from the organization’s human resources department to conduct the study within their workplace premises. The study participants were approached individually in their respective offices, and the data were collected through a paper survey. Furthermore, the respondents were also requested for their professional email addresses to have personal contact with them at any later stage of research. To endure impartiality in the responses, a cover letter was attached with a questionnaire that explained the purpose of the research and ensured the respondents that their response would remain anonymous and their participation in this study was completely voluntary. The flexibility of timing was also given to the respondents to fill out the questionnaire at their convenience. The participants were asked to give their feedback on statements about sleep patterns, deviant acts at the workplace, and job performance.

### 3.3. Description of Measures

The questionnaire was divided into four parts. The first part focused on the measure of sleep deprivation. The second part contained items for workplace deviance. The third part contained items for employee performance, and questions relating to the participants’ demographic information were asked in the fourth section. Considering the research techniques that were followed by Barnett [64] and Chen et al. [65], partial sleep deprivation was operationalized as a categorical variable. A total of 6 h was selected as the cut-off point. Consensus has emerged in several reviews of the sleep literature that sleeping six or fewer hours leads to cognitive impairment, whereas sleeping seven or more does not.

The measures asked the working mothers to indicate the number of hours that they sleep daily (especially the number of hours they slept the night before the survey). Specifically, the working mothers filled in the exact hours they were asleep using a series of check-boxes for each hour of the day. The individuals who slept six or fewer hours were coded as 1, representing sleep-deprived individuals. The respondents who had slept seven or more hours were coded as 0, indicating no sleep deprivation. Barnett [64] and Chen et al. [65] also have used the same approach for their studies. The authors used seven items from Bennett and Robinson’s [63] study for workplace deviance.

The participants were asked to indicate how often they had engaged in deviant behavior using a 5-point Likert scale from 1 = never to 5 = always. The sample items included: “I put little effort into my work” and “I have left my work for someone else to finish.” The employee performance was measured through a five-item scale that was drawn from the research of Dutch translation [66] of Podsakoff and MacKenzie’s [62]. The sample items were: “I always fulfill all the responsibilities required at my job”, “I always complete the duties specified in my job description”, and “I always meet all the formal performance requirements of my job”.

The response format ranged from “strongly disagree” (1) to “strongly agree” (5). The demographic variables in the study included the respondents’ age, nature of employment, length of service, level of education, and educational sector. See Table 2 for detailed demographic information of the respondents.

## 4. Data Analysis

The collected data were subjected to statistical and structural analyses. To ensure the appropriateness of the data for analysis, the authors investigated the missing values, and zero missing values were found. For detecting any aberrant values or outliers, two different steps were followed. In the first step, the cases outside the scale of 1–5 were eliminated. In the second step, the multivariate outliers were detected using the Mahalanobis distance measuring method. This process indicated 19 cases with multivariate outliers as their values were less than 0.001 and, therefore, were removed from the analysis. The final analysis was carried out with 340 cases/observations.

The researchers computed a Cronbach’s alpha value to analyze the reliabilities of the scales. The coefficient, which reflects homogeneity among items, varies from 0 to 1. The more the coefficient gets closer to 1, the better the reliability. However, the value of a coefficient of less than 0.60 is considered a poor one. According to Hair et al. [67], good reliability should produce a coefficient value of 0.70 or above. The analysis of Cronbach’s alpha for workplace deviance indicated a value of 0.849 and for employee performance indicated a value of 0.913, respectively. These results fully complied with the minimum 0.7 value that Hair et al. [67] recommended. A Pearson correlation coefficient was calculated to assess the relationship between sleep deprivation, workplace deviance, and employee performance. The prediction about the value of employee performance that was based on sleep deprivation and workplace deviance as the mediating variable was assessed through structural equation modeling (SEM) using IBM SPSS AMOS v.25 (IBM Corporation, Armonk, NY, USA).

To proceed further, the adequacy of the sample was assessed using the Kaiser–Meyer–Olkin (KMO) test. The KMO test presented a value of 0.877, which adequately complied with Kaiser and Rice’s [68] lowest suggested value of 0.6. The correlation matrix in Table 3 displays the correlation coefficient between the measured variables. A coefficient is considered significant if the *p*-value is less than 0.05. The Table 3 indicates that sleep deprivation has a significant positive correlation with workplace deviance (r = 0.386, *p* < 0.01) and has a negative relationship with employee performance (r = −0.354, *p* < 0.01). On the other side, workplace deviance is negatively correlated with employee performance (r = −0.475, *p* < 0.01).

### 4.1. Analyzing the Measurement and Structural Model

To test the stated hypotheses, the SEM technique was followed using IBM SPSS AMOS v.25. The SEM technique empowers researchers to build the hierarchy of latent constructs and eliminate the biases that are produced by measurement errors. The variance inflation factor (VIF) technique was used to examine the multi-collinearity issue, which indicated a value of 2.223 and matched with Hair et al.’s [67] suggested value of below 4.0 or less. To ensure the appropriateness of the data for measurement and structural analyses, Harman’s single factor test was used to examine the common method bias (CMB) issue, which indicated a value of 35.05% for a single factor. This value adequately complied with Podsakoff and MacKenzie’s [62] and Abbas and Sağsan’s [69] maximum single factor loading of 50% or less.

The authors performed confirmatory factor analysis (CFA) to test the latent variables’ link and determinants. CFA also helps the researchers to confirm the validity and one-dimensionality of the measurement model. As stated earlier, Cronbach’s alpha analysis for workplace deviance and employee performance were 0.849 and 0.913 values, respectively, and fully complied with the minimum 0.7 value that was recommended by Hair et al. [67]. To build reliability and validity, authors need to focus on composite reliability (CR), average variance explained (AVE), maximum shared squared variance (MSV), and average shared squared variance (ASV). The thresholds for these values are: reliability: CR > 0.7, convergent validity: CR > (AVE), AVE > 0.5, discriminant validity: MSV < AVE, ASV < AVE, (see the results of validities and reliabilities in Table 4). Based on the above-defined thresholds, it can be concluded that both constructs have composite reliability as their CR values are greater than 0.7 (workplace deviance = 0.851, employee performance = 0.951).

On the other hand, both constructs also meet the threshold criteria to establish discriminate validity as the identified values of given AVE are greater than the values of both MSV and ASV (workplace deviance = 0.453 > 0.251 & 0.210, employee performance = 0.830 > 0.251 & 0.206). Convergent validity is the only concern in the case of workplace deviance, as its given values do not perfectly meet the required threshold criteria, i.e., the value of CR should be greater than the value of AVE, which, in turn, should be greater than 0.5 (workplace deviance = 0.851 > 0.453 > 0.5). Although the value of CR is greater than the values of AVE, the value of AVE is very close to the standard of 0.5. Thus, the convergent validity of the constructs cannot be dismissed altogether. However, in the case of the “employee performance” construct, the value of CR is greater than the value of AVE, which is also greater than 0.5. As the given construct meets the desired threshold criteria to build the convergent validity thus, it can be concluded that the scale is valid and reliable.

As recommended by Kaynak [70], The authors examined the goodness of fit of the measurement and structural models using chi-square to the degree of freedom (CMIN/DF), TLI, CFI, and RMSEA indicators. As per Bagozzi and Yi [71], the maximum value for CMIN/DF is 3. Bentler and Bonett [72] and McDonald and Marsh [73] have suggested that the values of fit indices should be above 0.9. Moreover, Browne and Cudeck [74] proposed that the ideal value of RMSEA is lower than 0.08. The analysis of measurement and structural models presented X^2^/DF values 2.59 and 2.684, respectively, which is sufficiently less than the 3 value that is recommended by Bagozzi and Yi [71]. Similarly, the TLI and CFI values for both models were above 0.9, meeting the 0.9 or above ideal requirement by Browne and Cudeck [74]. Finally, the RMSEA values for measurement and structural models were 0.068 and 0.070, respectively, which significantly meets McDonald and Marsh’s [73] recommended value of less than 0.08.

### 4.2. Hypothesis Testing

**Figure 1:**
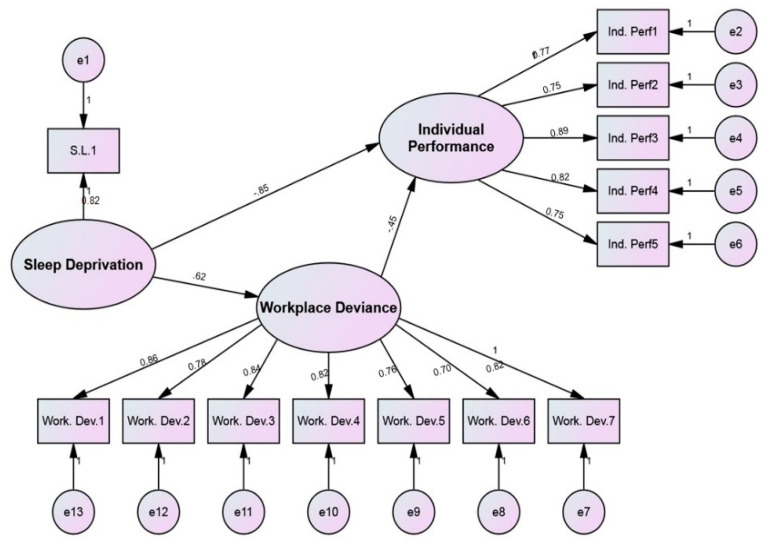
Measurement model.

The structural analysis indicated that sleep deprivation significantly negatively impacts mothers that are working in different educational institutions, with −0.8488 standardized estimates and 0.0000 *p*-values. Hence, the first hypothesis (H1), i.e., sleep deprivation has a significant negative impact on the job performance of working mothers, is accepted (see Figure 1). The analysis of the relationship between sleep deprivation and workplace deviance indicated a significant and positive relationship with a standardized estimated value of 0.6229 and 0.000 *p*-value. For this reason, the second hypothesis, (H2) sleep deprivation has a significant positive impact on workplace deviance, is also accepted. A significant negative relationship was identified while examining the link between workplace deviance and employee performance. The relationship between these two variables indicated a *p*-value of 0.0000 and a −0.4510 standardized estimate value. This leads to the acceptance of the third hypothesis, i.e., workplace deviance has a significant negative impact on the job performance of working mothers in the educational sector of Pakistan. See Table 5 for items loading and direct effect results.

The researchers analyzed the meditation effect of workplace deviance in the relationship between sleep deprivation and job performance through bootstrapping on 5000 bootstrap samples. The indirect effect analysis indicated significant results, as the 95% confidence interval does not include 0 (−0.4361 to −0.1645 in the case of WD). Since a “×” b “×” c (0.1077) is positive, it is a complimentary mediation (see Table 5). After examining the potential mediator, it can be concluded that workplace deviance is likely an important mediator. The computation of the confidence intervals for each indirect effect can be done in the usual way. The SPSS version of the macro has been used to bootstrap the indirect effects of sleep deprivation on employee performance. Table 6 presents a 95% confidence interval (percentile, BC, and BCa). In compliance with the results of the product-of-coefficients, it can be concluded that workplace deviance acts as a significant mediator between sleep deprivation and employee performance.

Further, the authors took age as the control variable. They examined whether it makes any difference in the relationship between sleep deprivation and individual performance and sleep deprivation and workplace deviance. The authors divided the data into two groups, i.e., mothers with below 35 age and mothers above 35 age. However, an insignificant difference was found in both cases with beta 0.118 and *p*-value 0.062 and 0.139 beta and 0.69 *p*-values, respectively. This means that if a person is under or above 35 years, their performance will be lower than normal life if they are deprived of sleep.

## 5. Discussion

The empirical results indicate that inadequate sleep in terms of quality and quantity is one of the main causes of working mothers’ deviant behavior and poor job performance. The findings relating to the sleep quality of working mothers and their job performance confirm the results of Swanson et al. [37] that poor sleep quality negatively affects employees’ attentiveness to the assigned tasks. It also validates Heider’s [75] idea, given in the attribution theory, that humans are motivated to assign causes to their actions and behaviors to external or internal factors.

Sleep deficiency also negatively impacts working mothers’ ability to complete the specified job in the prescribed manner. It diminishes their potential to comply with the changing demands of their current job. The ability to deal with the issues at hand is likewise low ebb. These working mothers need time to adapt to their new surroundings and learn to tolerate others’ opinions. Individuals are more prone to explosive emotional outbursts, harming their job relationships. Sleep-deprived working mothers also tend to put little effort into their work.

Additionally, it causes them to be less awake and concentrate. They find it more difficult to concentrate and pay attention, making them more easily confused. At the same time, this would impair their capacity to do activities that require logical reasoning or complex cognition. Sleepiness also hinders their judgment, making it more difficult to make the right decisions since they cannot appraise events and choose the appropriate conduct.

Moreover, they also tend to arrive late at work or take a longer break than the acceptable time limit. This, in turn, has a negative impact on an organization’s overall performance. This finding relates to Bultmann et al.’s [36] statement that sleep-deprived individuals tend to take more leaves than others. People who have such a negative attitude toward their professions are also less productive, resulting in a lower level of job satisfaction. Due to sleep deprivation, their professional and personal life are negatively impacted.

Thus, it can be said that sleep deprivation is believed to undermine the wellbeing of working mothers from different perspectives, i.e., emotionally, physically, and mentally, making it hard for them to keep their performance consistent and steady in the long run. This status of sleep deprivation can also force these working women to engage in activities that are not considered desirable and consequently severely affect their performance at the workplace.

### 5.1. Managerial Implications and Recommendations

Based on the findings of this study, it is recommended that the management arrange an array of rules about sleep just like every organization has concerning behaviors regarding smoking and provocation. It is essential to have rules about limited working hours, i.e., the company should not permit more than preferably eight hours of work a day and at the most, 10 hours successively. The government has a critical role in legislating and implementing such rules by coordinating with the companies.

Furthermore, offering new mothers paid maternity leaves with sufficient time serves as a very effective incentive. It will make them loyal and sincere to the company and is also an added financial benefit, improving the family’s status, and thus, dedication towards work. Female staff turnover will be at a minimum in those organizations that believe in providing maternity leave. This time off allows the mother to take care of herself and her baby. It eventually increases employee morale productivity and reduces their absenteeism at the workplace when moms would resume back to their work.

Adjustable or flexible working hours can also serve as a driving force for employed mothers to perform enthusiastically. The management can also provide on-site daycare facilities to bring their children and a caretaker to take care of the baby during office hours. The presence of such a facility would let the mother work at peace, knowing that she can visit her baby during breaks or free hours. However, as stated earlier, to translate implications into action, the role of government is pivotal.

### 5.2. Theoretical Contribution to Academicians

Over different periods, researchers have studied sleep deprivation and employee performance from different perspectives. However, the relationship between sleep deprivation and employee performance by considering the role of workplace deviance as a mediator, particularly in the context of working mothers in Pakistan, could not be found yet in the existing literature. Subsequently, researchers have observed a need to fill this research gap.

This study offers a significant contribution to the organizational literature by integrating research from the social, psychological, sleep, and neurocognitive literature to develop and test a theoretically- and empirically driven model of the effects of sleep deprivation on the performance of working mothers. Moreover, this study is not only contributing to the literature by concurrently studying the direct effects of sleep deprivation on the performance of working mothers, but also takes into account other psychological factors, such as how sleep deprivation can make an employee indulge in deviant acts, such as are these working mothers coming late to their work without taking permission or are they leaving their work for someone else to finish, which in turn can also ultimately deteriorate their performance at the workplace.

Additionally, as with the changing trends, Pakistani culture is now promoting gender diversity to bring more creativity and diverse thinking within the organizational settings. Hence, the number of working mothers in the workplace is increasing rapidly. Undoubtedly, these working mothers face many challenges in coping with their professional and personal duties. These working mothers need to strike a balance between their personal and professional lives, which has been a matter of growing concern for managers of the organizations. Therefore, the current research’s findings will be a significant value addition to the existing literature on sleep deprivation, which can be helpful for the managers to uplift the performance of working mothers at the workplace. All these above-discussed factors have distinguished this study from the previous empirical research, which has been conducted in the past decade. Hence, it can be summed up that this empirical study will give more in-depth insights, mainly for the educational sector of Pakistan.

Moreover, as discussed above, in recent times, women have become more career-oriented and are more actively taking part in jobs across the globe. Thus, a considerable shift has been observed in women’s attachment in serving different positions for which they are paid within the organizations. This highly popular trend has brought two questions of concern: (1) should young children’s mothers be given the liberty and permission to work in the corporate world? and (2) if they do so, who will be accountable for ensuring that the children back at home are being taken care of? Hence, it follows that the management of the organizations where the mothers are employed should be considerate enough to facilitate them in the best possible manner. Therefore, they can have a balanced healthy, and happy life. To provide them with a work-life balance and encourage more mothers to continue working after a baby, some parent-friendly policies that serve as a support system for the mother post-pregnancy are a must. This will eventually lead to greater job satisfaction, productivity, and enhanced performance at the workplace.

### 5.3. Research Limitations and Future Recommendations

Despite the unwavering and concrete efforts of the researcher to provide enough proof and evidence to support the study, the need to highlight certain limitations remains a necessity so that they can be avoided in future research.

The targeted population of this particular study is the part of a selected segment (educational sector), which, in turn, is a part of the service sector that is located in Pakistan. Thus, it should be borne in mind that the results may not be generalized to all services organizations of Pakistan. Although, the impact of sleep deprivation on the job performance of working mothers would be different from the ones in other sectors due to their environmental and contextual differences. The basic difference lies in the demand of profession, i.e., eternal, post-work hours engagement with various stakeholders (co-teachers, parents, school management, and students) coupled with pressure to timely complete the administrative tasks such as lesson planning, maintenance of attendance, etc. makes teaching a 24/7 job [76]. Whereas, in other sectors, the workload ends with the working day. We assume that the case of other segments of service sector employees is marginally better than the education sector employees. Hence, we can assume that there wouldn’t be such severely negative results. However, to understand the phenomenon in other segments in the service sector, a concentrated study on the phenomenon’s impact is needed. Therefore, to further study the relationship between sleep-deprived individuals and their performance, other service industry segments can also be considered in future research. These sectors may include health, transportation, construction, tourism, etc. On the other side, the population for the current research study comprised of working mothers (female staff), so in the future, along with female staff, male staff can also be considered for the more generalized result.

Another limitation of this research is that only one variable has been taken into account as a mediator to establish a relationship between sleep deprivation and employee performance, which is workplace deviance. A few other variables can also be researched to understand better the correlation and association between sleep deprivation and employee performance. These dimensions have also been identified in the past literature, i.e., mood, stress, emotions, physical health, mental health, etc. These can also be included in future research as mediators. Additionally, the future authors should consider other variables such as socioeconomic status, the number of children, single-parent status, etc., that could affect the outcomes.

Additionally, future researchers can extend the scope of the given model by studying the causes of sleep deprivation among working mothers. However, to bring more insightful results, the issue of attribution needs to be elaborated to reflect how employees will attribute their sleep deprivation due to caring responsibilities at home to the institutions where they work, leading to deviance and subsequently reducing their performance. Therefore, it should be kept in mind that future researchers should aim to use objective-based measures to gain more reliable and valid results.

This study focuses only on employees as the source of data for analysis. Any discrepancy in their feedback may manipulate the measures, leading to imprecise findings. Thus, to mitigate such discrepancy, future studies are suggested to be performed by collecting data from diverse sources. Moreover, the given constructs are measured based on an individual’s perception and can serve as deterrence in achieving valid results. For this reason, future studies should emphasize objective-based measurements more than subjective measures since they are more trustworthy and accurate.

Moreover, in the current research, the authors report that sleep duration depends on the number of hours that are spent sleeping in the previous days, particularly the night before the survey. Although, the consensus has emerged in several reviews of the sleep literature that sleeping six or fewer hours leads to cognitive impairment, whereas sleeping seven or more does not. Many renowned researchers have used the given scale in their studies, such as Barnett [64] and Chen et al. [65] also have used the same approach for their studies. However, this is also confusing since sleep deprivation the night before the survey may alter responses to survey questions. Therefore, future researchers suggest assessing the average sleep duration rather than the sleep duration just the night before the survey as poor job performance is usually related to long-term sleep deprivation. They are also required to have more details in the survey such as average bedtime, average wake up time, and average sleep duration will probably provide more details.

The fairly low number of respondents is a further limitation of the study, even with an acceptable minimum number (300). Even though the results that were found are significant and considerable, the small size of the sample is expected to lack the component of generalizability and statistical power. This issue can be rightly addressed by increasing the size of the researchers’ sample in future research.

## 6. Conclusions

This research is focused on examining the impact of sleep deprivation on the job performance of mothers that are working in primary, secondary, and higher educational institutions in Pakistan. Women’s roles in the workplace have evolved worldwide due to economic and social factors. Working mothers are under a lot of pressure to advance in their careers while still maintaining a high level of involvement in their home life. Due to ever-increasing professional and family commitments, they have less time for themselves, resulting in sleep deprivation which decreases productivity in the workplace. Therefore, the conceptual framework of this study has broadly been assorted into three categories, namely situational, psychological, and behavioral mechanisms. As the situational antecedent, sleep deprivation is positively correlated with workplace deviance, which makes up the psychological mechanism. This factor, in turn, negatively affects the behavioral outcome, called individual performance at the workplace.

## Figures and Tables

**Table 1:** Response rate (pilot and main study).

No	Educational Sector	Sample	Responses	Response %
**Pilot Study**				
1	Schools	28	25	89
2	Colleges	18	17	94
3	Universities	14	11	78
Total	Educational Sectors	60	53	88
**Main Study**				
1	Schools	210	201	96
2	Colleges	120	108	90
3	Universities	72	68	97
Total	Educational Sectors	402	377	94

**Table 2:** Demographic characteristics of the respondents (*n* = 377).

Variables		Frequency	Percentage
**Age**	Up to 35 Years	138	37
	Above 35 Years	241	63
**Educational level**	Undergraduates	0	0
	Graduates	168	45
	M.Phil.	113	29
	PhD	98	26
**Length of Service**	01–05	58	15
	06–10	192	51
	>10	129	34
**Nature of Employment**	Contractual	27	7
	Permanent	352	93

**Table 3:** Correlation matrix.

Variable	Sleep Deprivation	Workplace Deviance	Employee Performance
Sleep Deprivation	-		
Workplace Deviance	0.386	-	
Employee Performance	−0.354	−0.475	-

**Table 4:** Convergent and discriminant validity and construct reliability.

	CR	AVE	MSV	ASV	Workplace Deviance	Employee Performance
**Workplace Deviance**	0.851	0.453	0.251	0.210	0.673	
**Employee Performance**	0.951	0.830	0.251	0.206	−0.501	0.911

**Table 5:** Testing of Hypotheses and Loadings (Direct Effect).

	Number of Items	Loading	Standardized Estimates	*p*-Value	Decision
**Workplace Deviance**	7	0.70–0.86			
**Individual Performance**	5	0.75–0.89			
**H1: Sleep Dep. > Ind. Per.**	-	-	−0.848	0.0000	Accepted
**H2: Sleep Dep. > Work. Dev.**	-	-	0.629	0.0000	Accepted
**H3: Work. Dev. > Ind. Per.**	-	-	−0.451	0.0000	Accepted

Sleep Dep.: Sleep Deprivation; Ind. Per.: Individual Performance; Work. Dev.: Workplace Deviance.

**Table 6:** Analysis of mediation effect with 95% confidence interval.

Variables	Point Estimates	Product of Coefficients		Bootstrapping					
				BCa 95% CI		Percentile 95% CI		BC 95% CI	
		SE	Z	Lower	Upper	Lower	Upper	Lower	Upper
**Mean_WD**	−0.2809	0.0611	−4.5981	−0.4361	−0.1645	−0.4140	−0.1612	−0.4345	−0.1702
**Total**	−0.4652	0.0744	6.2551	−0.6366	−0.3218	−0.6244	−0.3186	−0.6373	−0.3284

Mean WD: Workplace Deviance; Analysis of Mediation Effect with 95% Confidence Interval on 5000 bootstrap samples.

## Data Availability

Data can be made available to individuals upon valid justification.

## References

[1] Hwang J., Abbas J., Joo K., Choo S.-W., Hyun S.S. (2022). The Effects of Types of Service Providers on Experience Economy, Brand Attitude, and Brand Loyalty in the Restaurant Industry. Int. J. Environ. Res. Public Health.

[2] Imran M., Abbas J. (2020). The Role Of Strategic Orientation In Export Performance Of China Automobile Industry. Handbook of Research on Managerial Practices and Disruptive Innovation in Asia.

[3] Pinto L.H., He K. (2018). In the Eyes of the Beholder: The Influence of Academic Performance and Extracurricular Activities on the Perceived Employability of Chinese Business Graduates. Asia Pac. J. Hum. Resour..

[4] Habib M., Abbas J., Noman R. (2019). Are Human Capital, Intellectual Property Rights, and Research and Development Expenditures Really Important for Total Factor Productivity? An Empirical Analysis. Int. J. Soc. Econ..

[5] Mahmood H.K., Hussain F., Mahmood M., Kumail R., Abbas J. (2020). Impact of E-Assessment at Middle School Students’ Learning—An Empirical Study at USA Middle School Students. Int. J. Sci. Eng. Res..

[6] Makkonen P. (2017). The Employability of Newcomer Self-Initiated Expatriates in China: An Employers’ Perspective. Asia Pac. J. Hum. Resour..

[7] Abbas J., Muzaffar A., Mahmood H.K., Ramzan M.A., Rizvi S.S.U.H. (2014). Impact of Technology on Performance of Employees (A Case Study on Allied Bank Ltd., Pakistan). World Appl. Sci. J..

[8] Shakoor F., Fakhar A., Abbas J. (2021). Impact of Smartphones Usage on the Learning Behaviour and Academic Performance of Students: Empirical Evidence from Pakistan. Int. J. Acad. Res. Bus. Soc. Sci..

[9] Ahsan M.U., Nasir M., Abbas J. (2020). Examining the Causes of Plastic Bags Usages and Public Perception about Its Effects on the Natural Environment. Int. J. Acad. Res. Bus. Soc. Sci..

[10] Xie Z., Liu X., Najam H., Fu Q., Abbas J., Comite U., Cismas L.M., Miculescu A. (2022). Achieving Financial Sustainability through Revenue Diversification: A Green Pathway for Financial Institutions in Asia. Sustainability.

[11] Mahmood H.K., Hashmi M.S., Shoaib D.M., Danish R., Abbas J. (2014). Impact of TQM Practices on Motivation of Teachers in Secondary Schools Empirical Evidence from Pakistan. J. Basic Appl. Sci. Res..

[12] Abbas J. (2020). Service Quality in Higher Education Institutions: Qualitative Evidence from the Students’ Perspectives Using Maslow’s Hierarchy of Needs. Int. J. Qual. Serv. Sci..

[13] Shahzad M., Ying Q., Ur Rehman S., Zafar A., Ding X., Abbas J. (2020). Impact of Knowledge Absorptive Capacity on Corporate Sustainability with Mediating Role of CSR: Analysis from the Asian Context. J. Environ. Plan. Manag..

[14] Abbas J., Alturki U., Habib M., Aldraiweesh A., Al-Rahmi W.M. (2021). Factors Affecting Students in the Selection of Country for Higher Education: A Comparative Analysis of International Students in Germany and the UK. Sustainability.

[15] Safdar B., Habib A., Amjad A., Abbas J. (2020). Treating Students as Customers in Higher Education Institutions and Its Impact on Their Academic Performance. Int. J. Acad. Res. Progress. Educ. Dev..

[16] Li W. (2018). Research on the Innovative Development Mode of Quality Education of College Students Based on the Perspective of Human Resource Management. Educ. Sci. Theory Pract..

[17] Abbas J. (2020). Impact of Total Quality Management on Corporate Green Performance through the Mediating Role of Corporate Social Responsibility. J. Clean. Prod..

[18] NSF Sleep in America. http://www.sleepfoundation.org.

[19] Schmitt A., Belschak F.D., Den Hartog D.N. (2017). Feeling Vital after a Good Night’s Sleep: The Interplay of Energetic Resources and Self-Efficacy for Daily Proactivity. J. Occup. Health Psychol..

[20] Kelley H.H., Michela J.L. (1963). Attribution Theory and Research. Annu. Rev. Psychol..

[21] Weiner B. (1985). An Attributional Theory of Achievement Motivation and Emotion. Psychol. Rev..

[22] Abbas J. (2020). HEISQUAL: A Modern Approach to Measure Service Quality in Higher Education Institutions. Stud. Educ. Eval..

[23] Gong Y., Yang S., Ma H., Ge M. (2018). Fuzzy Regression Model Based on Incentre Distance and Application to Employee Performance Evaluation. Int. J. Fuzzy Syst..

[24] Kumari K., Usmani S., Siddiqi S.J., Husain J. (2016). The Effect of Sleep Deprivation on the Job Performance of Working Mothers. J. Bus. Stud..

[25] Andries P., Stephan U. (2019). Environmental Innovation and Firm Performance: How Firm Size and Motives Matter. Sustainability.

[26] Kazmi S.J.A., Abbas J. (2021). Examining the Impact of Industry 4.0 on Labor Market in Pakistan. Handbook of Smart Materials, Technologies, and Devices.

[27] Abbas J., Kumari K. (2021). Examining the Relationship between Total Quality Management and Knowledge Management and Their Impact on Organizational Performance. J. Econ. Adm. Sci..

[28] Litwiller B., Snyder L.A., Taylor W.D., Steele L.M. (2017). The Relationship between Sleep and Work: A Meta-Analysis. J. Appl. Psychol..

[29] Weinger M., Ancoli-Israel S. (2012). Sleep Deprivation and Clinical Performance. JAMA.

[30] McKibben J.B., Fullerton C.S., Ursano R.J., Reissman D.B., Kowalski-Trakofler K., Shultz J.M., Wang L. (2010). Sleep and Arousal as Risk Factors for Adverse Health and Work Performance in Public Health Workers Involved in the 2004 Florida Hurricane Season. Disaster Med. Public Health Prep..

[31] Sivertsen B., Björnsdóttir E., ØVerland S., Bjorvatn B., Salo P. (2013). The Joint Contribution of Insomnia and Obstructive Sleep Apnoea on Sickness Absence. J. Sleep Res..

[32] Abbas J. (2020). Impact of Total Quality Management on Corporate Sustainability through the Mediating Effect of Knowledge Management. J. Clean. Prod..

[33] Barnes C.M., Hollenbeck J.R. (2009). Sleep Deprivation and Decision-Making Teams: Burning the Midnight Oil or Playing with Fire?. Acad. Manag. Rev..

[34] Rajaratnam S.M., Barger L.K., Lockley S.W., Shea S.A., Wang W., Landrigan C.P., O’Brien C.S., Qadri S., Sullivan J.P., Cade B.E. (2011). Sleep Disorders, Health, and Safety in Police Officers. JAMA.

[35] Sirois F.M., van Eerde W., Argiropoulou M.I. (2015). Is Procrastination Related to Sleep Quality? Testing an Application of the Procrastination–Health Model. Cogent Psychol..

[36] Bültmann U., Nielsen M.B.D., Madsen I.E., Burr H., Rugulies R. (2012). Sleep Disturbances and Fatigue: Independent Predictors of Sickness Absence? A Prospective Study among 6538 Employees. Eur. J. Public Health.

[37] Swanson L.M., Arnedt J.T., Rosekind M.R., Belenky G. (2011). Sleep Disorders and Work Performance: Findings from the 2008 National Sleep Foundation Sleep in America Poll. J. Sleep Res..

[38] Diestel S., Rivkin W., Schmidt K.H. (2015). Sleep Quality and Self-Control Capacity as Protective Resources in the Daily Emotional Labor Process: Results from Two Diary Studies. J. Appl. Psychol..

[39] Dobos N. (2017). What’s So Deviant about Production Deviance? The Ethics of “Withholding Effort” in the Workplace. Soc. Theory Pract..

[40] Bardos K.S., Ertugrul M., Gao L.S. (2020). Corporate Social Responsibility, Product Market Perception, and Firm Value. J. Corp. Financ..

[41] Castro-González S., Bande B., Kimura T. (2019). How and When Corporate Social Responsibility Affects Salespeople’s Organizational Citizenship Behaviors?: The Moderating Role of Ethics and Justice. Corp. Soc. Responsib. Environ. Manag..

[42] Khan S.M., Abbas J. (2022). Mindfulness and Happiness and Their Impact on Employee Creative Performance: Mediating Role of Creative Process Engagement. Think. Ski. Creat..

[43] Utkarsh V., Ravindra T., Ananta N. (2019). Workplace Deviance: A Conceptual Framework. Int. J. Recent Technol. Eng..

[44] Kühnel J., Bledow R., Feuerhahn N. (2016). When Do You Procrastinate? Sleep Quality and Social Sleep Lag Jointly Predict Self-regulatory Failure at Work. J. Organ. Behav..

[45] Barnes C.M. (2012). Working in Our Sleep: Sleep and Self-Regulation in Organizations. Organ. Psychol. Rev..

[46] Bennett R.J., Robinson S.L., Greenverg J. (2013). The Past, Present, and Future of Workplace Deviance Research. Organizational Behavior: The State of the Science.

[47] Kühnel J., Zacher H., De Bloom J., Bledow R. (2017). Take a Break! Benefits of Sleep and Short Breaks for Daily Work Engagement. Eur. J. Work. Organ. Psychol..

[48] Abbas J., Muzaffar A., Shoaib M., Mahmood H.K. (2014). Do Business Schools Really Fulfill Industry Requirements? An Investigation of Industrial Performance of Business Graduates. World Appl. Sci. J..

[49] Dunlop P.D., Lee K. (2004). Workplace Deviance, Organizational Citizenship Behavior, and Business Unit Performance: The Bad Apples Do Spoil the Whole Barrel. J. Organ. Behav. Int. J. Ind. Occup. Organ. Psychol. Behav..

[50] Uehli K., Mehta A.J., Miedinger D., Hug K., Schindler C., Holsboer-Trachsler E., Leuppi J.D., Künzli N. (2014). Sleep Problems and Work Injuries: A Systematic Review and Meta-Analysis. Sleep Med. Rev..

[51] Åkerstedt T., Fredlund P., Gillberg M., Jansson B. (2002). A Prospective Study of Fatal Occupational Accidents–Relationship to Sleeping Difficulties and Occupational Factors. J. Sleep Res..

[52] Kuhl M.R., Cunha J.C.D., Macaneiro M.B., Cunha S.K. (2016). Relationship between Innovation and Sustainable Performance. Int. J. Innov. Manag..

[53] Mawritz M.B., Mayer D.M., Hoobler J.M., Wayne S.J., Marinova S.V. (2012). A Trickle-down Model of Abusive Supervision. Pers. Psychol..

[54] Abbas J., Mahmood H.K., Hussain F. (2015). Information Security Management for Small and Medium Size Enterprises. Sci. Int..

[55] Appelbaum S.H., Deguire K.J., Lay M. (2005). The Relationship of Ethical Climate to Deviant Workplace Behaviour. Corp. Gov. Int. J. Bus. Soc..

[56] Rotundo M., Sackett P.R. (2002). The Relative Importance of Task, Citizenship, and Counterproductive Performance to Global Ratings of Job Performance: A Policy-Capturing Approach. J. Appl. Psychol..

[57] Abbas J., Sagsan M. (2019). Identification of Key Employability Attributes and Evaluation of University Graduates’ Performance: Instrument Development and Validation. High. Educ. Ski. Work-Based Learn..

[58] Colbert A.E., Mount M.K., Harter J.K., Witt L.A., Barrick M.R. (2004). Interactive Effects of Personality and Perceptions of the Work Situation on Workplace Deviance. J. Appl. Psychol..

[59] Litzky B.E., Eddleston K.A., Kidder D.L. (2006). The Good, the Bad, and the Misguided: How Managers Inadvertently Encouraged Deviant Behaviors. Acad. Manag. Perspect..

[60] Chiu S., Peng J. (2008). The Relationship between Psychological Contract Breach and Employee Deviance: The Moderating Role of Hostile Attributional Style. J. Vocat. Behav..

[61] Bennett R.J., Robinson S.L. (2000). Development of a Measure of Workplace Deviance. J. Appl. Psychol..

[62] Podsakoff P.M., MacKenzie S.B. (1989). A Second Generation Measure of Organizational Citizenship Behavior.

[63] Sekaran U. (2003). Research Methods for Business: A Skill-Building Approach.

[64] Barnett K.J. (2008). The Effects of a Poor Night Sleep on Mood, Cognitive, Autonomic and Electrophysiological Measures. J. Integr. Neurosci..

[65] Chen J.H., Gill T.M., Prigerson H.G. (2005). Health Behaviors Associated with Better Quality of Life for Older Bereaved Persons. J. Palliat. Med..

[66] Van Yperen N.W., de Jong A. (1997). Is Een Tevreden Werknemer Ook Een Productieve Werknemer. Gedrag Organ..

[67] Hair J.F., Anderson R.E., Tatham R.L., Black W.C. (2010). Multivariate Data Analysis.

[68] Kaiser H.F., Rice J. (1974). Little Jiffy, Mark Iv. Educ. Psychol. Meas..

[69] Abbas J., Sağsan M. (2019). Impact of Knowledge Management Practices on Green Innovation and Corporate Sustainable Development: A Structural Analysis. J. Clean. Prod..

[70] Kaynak H. (2003). The Relationship between Total Quality Management Practices and Their Effects on Firm Performance. J. Oper. Manag..

[71] Bagozzi R.R., Yi Y. (1988). On the Evaluation of Structural Equation Models. J. Acad. Mark. Sci..

[72] Bentler P.M., Bonett D.G. (1980). Significance Tests and Goodness of Fit in the Analysis of Covariance Structures. Psychol. Bull..

[73] McDonald R.P., Marsh H.W. (1990). Choosing a Multivariate Model: Noncentrality and Goodness of Fit. Pschological Bull..

[74] Browne M.W., Cudeck R. (1992). Alternative Ways of Assessing Model Fit. Sociol. Methods Res..

[75] Heider F. (1958). The Psychology of Interpersonal Relations.

[76] Skaalvik E.M., Skaalvik S. (2018). Job Demands and Job Resources as Predictors of Teacher Motivation and Well-Being. Soc. Psychol. Educ..

